# Metabolically Active Zones Involving Fatty Acid Elongation Delineated by DESI-MSI Correlate with Pathological and Prognostic Features of Colorectal Cancer

**DOI:** 10.3390/metabo13040508

**Published:** 2023-03-31

**Authors:** Martin Kaufmann, Natasha Iaboni, Amoon Jamzad, David Hurlbut, Kevin Yi Mi Ren, John F. Rudan, Parvin Mousavi, Gabor Fichtinger, Sonal Varma, Antonio Caycedo-Marulanda, Christopher J. B. Nicol

**Affiliations:** 1 Department of Surgery, Kingston Health Sciences Centre, Kingston, ON K7L 2V7, Canada; martin.kaufmann@queensu.ca (M.K.); john.rudan@kingstonhsc.ca (J.F.R.);; 2 Gastrointestinal Diseases Research Unit, Kingston Health Sciences Center, Kingston, ON K7L 2V7, Canada; 3 Department of Pathology and Molecular Medicine, Queen’s University and Kingston Health Sciences Centre, Kingston, ON K7L 3N6, Canada; 15ni8@queensu.ca (N.I.); david.hurlbut@kingstonhsc.ca (D.H.); kevin.ren@kingstonhsc.ca (K.Y.M.R.);; 4 School of Computing, Queen’s University, Kingston, ON K7L 2N8, Canada; a.jamzad@queensu.ca (A.J.); mousavi@queensu.ca (P.M.); fichting@queensu.ca (G.F.); 5 Orlando Health Colon and Rectal Institute, Orlando, FL 32806, USA; 6 Queen’s Cancer Research Institute, Division of Cancer Biology and Genetics, Kingston, ON K7L 3N6, Canada

**Keywords:** colorectal cancer, desorption electrospray ionization mass spectrometry imaging, histopathology, prognostic markers, biopsy, long chain fatty acids, lymphovascular invasion

## Abstract

Colorectal cancer (CRC) is the second leading cause of cancer deaths. Despite recent advances, five-year survival rates remain largely unchanged. Desorption electrospray ionization mass spectrometry imaging (DESI) is an emerging nondestructive metabolomics-based method that retains the spatial orientation of small-molecule profiles on tissue sections, which may be validated by ‘gold standard’ histopathology. In this study, CRC samples were analyzed by DESI from 10 patients undergoing surgery at Kingston Health Sciences Center. The spatial correlation of the mass spectral profiles was compared with histopathological annotations and prognostic biomarkers. Fresh frozen sections of representative colorectal cross sections and simulated endoscopic biopsy samples containing tumour and non-neoplastic mucosa for each patient were generated and analyzed by DESI in a blinded fashion. Sections were then hematoxylin and eosin (H and E) stained, annotated by two independent pathologists, and analyzed. Using PCA/LDA-based models, DESI profiles of the cross sections and biopsies achieved 97% and 75% accuracies in identifying the presence of adenocarcinoma, using leave-one-patient-out cross validation. Among the m/z ratios exhibiting the greatest differential abundance in adenocarcinoma were a series of eight long-chain or very-long-chain fatty acids, consistent with molecular and targeted metabolomics indicators of de novo lipogenesis in CRC tissue. Sample stratification based on the presence of lympovascular invasion (LVI), a poor CRC prognostic indicator, revealed the abundance of oxidized phospholipids, suggestive of pro-apoptotic mechanisms, was increased in LVI-negative compared to LVI-positive patients. This study provides evidence of the potential clinical utility of spatially-resolved DESI profiles to enhance the information available to clinicians for CRC diagnosis and prognosis.

## 1. Introduction

Colorectal cancer (CRC) is the third leading cause of new cancer cases, and the second leading cause of cancer-related deaths for women and men combined, both globally and among Canadians [1,2]. In 2022, an estimated 1.9 million cases of CRC and 935,000 deaths occurred globally [3]. In Canada 24,300 new cases, and 9400 CRC-related deaths were reported [1]. Both nonmodifiable and modifiable factors, including age, genetics, biological sex, and environmental and lifestyle factors such as obesity, may influence individual CRC risk, tumour aggressiveness, and treatment outcomes [4,5]. The great majority of colorectal tumours are adenocarcinomas that arise from the epithelial cells of the colorectal mucosa [6]. Histopathological assessment of a given tumour forms the current basis for diagnosis and clinical management. Pathology assessment includes an evaluation of tumour spread (depth of mural invasion; regional lymph node involvement; presence or absence of distant metastasis) that defines tumour stage and other histopathological parameters that inform prognosis (such as degree of differentiation [grade], lymphovascular and/or perineural invasion, host inflammatory reaction) or predict response to therapy (such as mismatch repair status). Molecular biomarkers are increasingly utilized in adjuvant oncologic treatment decisions [7,8,9]. Currently, there are four consensus molecular subtypes (CMS) of CRC that may influence a patient’s prognosis and treatment options; CMS1, CMS2, CMS3, and CMS4 [10,11]. The CMSs are based upon a variety of factors including mutational status, immune activation, and metabolic dysregulation described in detail elsewhere [8]. Despite advances in early screening and therapeutic options that have reduced mortality in those over the age of 50, the alarming increase over the last decade in CRC death rates among patients younger than 50 years of age may be due to increasing obesity levels and body mass indices in those populations [3,12]. Even so, the impact of BMI on increased CRC risk is consistent across all molecular subtypes [12], suggesting more work is still needed to improve our understanding of individual CRC risk and response to treatment to improve clinical outcomes.

While the genetic and molecular aspects of CRC are heavily studied, interrogation of CRC tissue using metabolomic approaches may offer new ways to identify diagnostic, prognostic and predictive biomarkers. Mass spectrometry-based methods allow the characterization of cell phenotype and metabolic activity, including pathways that trigger or promote malignant transformation. CRC patient plasma, serum, and tissues have been studied using a variety of mass spectrometry modalities. One study identified dysregulated amino acid and triacylglyceride (TAG) metabolism in CRC [13,14]. Shen et al. also found CRC tissues exhibit elevated glutathione metabolism in order to counter increasing oxidative stress [15]. In addition, studies exploring the relationship between the metabolome and microbiota in CRC have been reported [3,16]. Desorption electrospray ionization-mass spectrometry imaging (DESI-MSI) is a nondestructive metabolomic approach that allows for the profiling of small molecules directly from the surfaces of fresh frozen tissue sections, without the need for additional tissue processing other than cryosectioning, as described elsewhere [17,18]. As such, the spatial location of detected m/z ratios is retained, allowing for ‘gold standard’ histological validation of the same slide following DESI analysis and hematoxylin and eosin (H and E) staining.

To assess metabolic alterations in CRC and their ability to correlate with histology annotations and known biomarkers, we performed a feasibility study of human CRC patient samples containing malignant and adjacent non-neoplastic tissue along with their accompanying simulated biopsies using DESI. We aimed to characterize metabolite profiles associated with adenocarcinoma and other tissue types in the colon and rectum, as well as explore the reproducibility and clinical utility of these profiles across patients using multivariate statistics. As certain differentially abundant m/z ratios observed in this study using DESI correspond to ions identified in other mass spectrometry analyses of colorectal tissue, we discuss possible metabolic perturbations that are consistent with known pathological and prognostic attributes in CRC.

## 2. Materials and Methods

### 2.1. Clinical Samples

Human CRC tissue samples were obtained prospectively from 10 randomly-selected patients undergoing surgical resection at Kingston Health Sciences Center in 2020–2021 (Table 1). The study protocol was approved by the Health Sciences Research Ethics Board of Queen’s University (File No. 6018391). Fresh CRC tissue for DESI-MSI analysis was sampled from the resection specimen immediately after receipt in the pathology lab from the operating room (cold ischemic time less than 20 min) and only when this additional sampling did not affect the standard protocol for clinical diagnostic purposes. Tissue was sampled from each of the 10 specimens using two methods: (1) a representative cross section of the tumour with adjacent non-neoplastic tissue containing all layers of the full thickness of the bowel wall and including colorectal adenocarcinoma, mucosa, submucosa, muscularis propria (smooth muscle), and serosa, and (2) biopsy samples of tumour and non-neoplastic mucosa obtained using an endoscopic biopsy forceps as a way to simulate the standard-of-care endoscopic biopsy procedure. Tissues were embedded in an optimal cutting temperature (OCT) medium, flash-frozen, and cryosectioned to a 10 µm thickness on uncharged slides. The slides were stored at −80 °C until DESI analysis.

### 2.2. DESI Mass Spectrometry Imaging Analysis

**Figure 1:**
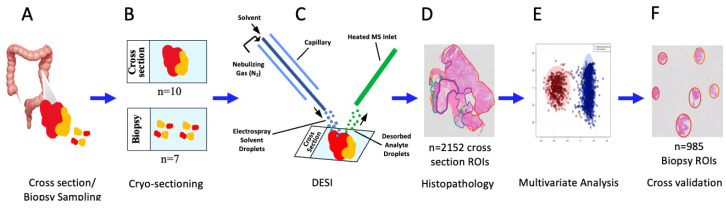
Experimental Overview. (**A**) Fresh frozen colorectal tissue containing adenocarcinoma and adjacent non-neoplastic tissue were obtained from 10 colorectal adenocarcinoma patients. Tissue cross sections and simulated biopsies were sampled for analysis. (**B**) Slides for each sample were prepared by cryosectioning to a thickness of 10 µm. (**C**) Each slide was analyzed by DESI, in negative ionization mode. (**D**) Slides were then annotated by pathologists to identify the regions containing adenocarcinoma, benign mucosa, submucosa, smooth muscle, serosa, and inflammatory cells. Approximately fifty 9-pixel regions of interest (ROIs) per pathologically-annotated region were plotted. (**E**) Two multivariate models based on PCA/LDA were created using the m/z 50–1500 mass range with different class labels including; Adenocarcinoma versus all non-neoplastic tissue regions and a 6-class model where ROIs from each pathologically-annotated region were labelled as individual classes. (**F**) Leave-one-patient-out cross validation was performed in order to identify adenocarcinoma in the tissue cross sections and biopsies, as well as pathologically annotated regions in the cross sections.

Slides containing tumour cross sections and simulated biopsies were subjected to mass spectrometry imaging analysis in negative ionization mode using a Xevo G2-XS time-of-flight mass spectrometer (Waters) fitted with a 2D DESI source (Prosolia); equipped with a high-performance sprayer and heated inlet transfer line (Waters). A solvent of 95% (v/v) methanol/water containing 0.05 ng/µL of leucine-enkephalin was delivered to the sprayer via a syringe pump at a rate of 2.25 µL/min (Harvard Apparatus). All solvents were of LC-MS Optima grade (Fisher). Mass spectral imaging data was acquired over the m/z 50–1500 range at a resolution of 100 × 100 µm, at a rate of 175 µm/s. Other mass spectrometry and DESI settings include source temperature of 100 °C; capillary voltage of 0.57 kV; nebulizing N_2_ gas of approximately 15 psi (Figure 1).

### 2.3. Pathology Analysis and Slide Annotation

The electronic pathology database at our institution was reviewed for all cases, after DESI analysis. Recorded elements included tumour location (cecum, ascending colon, transverse colon, descending colon, sigmoid colon, and rectum), histologic grade, tumour size, tumour (T) stage, resection margin status (positive or negative), lymph nodes harvested (number), lymph nodes positive for metastatic tumour (number), nodal (N) stage, presence or absence of lymphovascular invasion (LVI), and mismatch repair status (intact or defcient) as assessed by immunohistochemistry. Following DESI profiling, tissue sections were stained using hematoxylin and eosin and whole slide digital images were annotated to delineate tumour versus nontumour regions (including different tissue layers and types) to correlate with DESI metabolic profiles (Figure 1).

### 2.4. Region of Interest Selection and Peak Alignment

High-Definition Imaging (HDI) software version 1.6 (Waters) was used to generate mass spectral images based on the top 2000 most intense m/z ratios that were mass-shift corrected to the leucine-enkephalin peak (m/z 554.2615, [M-H]^−^), and intensities were normalized to the total ion current (TIC). Gold standard histopathology annotations were used to guide the selection of nine-pixel regions of interest (ROIs). Approximately 55 ROIs were sampled for each pathologically-annotated region defined as adenocarcinoma cross section (*n* = 563 ROIs) biopsy (*n* = 551), benign mucosa cross section (*n* = 437) biopsy (*n* = 309), submucosa (*n* = 384), smooth muscle (*n* = 384), serosa (*n* = 238), and inflammatory cells cross section (*n* = 141) biopsy (*n* = 116). To align the m/z ratios among samples, a custom code was developed in MATLAB that uses kernel-smoothing density estimation with a bandwidth of m/z 0.003 to cluster and bin m/z ratios among all scanned tissues. Mass bins with a 0.003 Da width are defined as the m/z corresponding to the centre of the bin. The peak list was filtered to include only the m/z ratios that were present in 40% of the slides (*n* > 8). The final peak list consisted of 1941 m/z ratios among all cross sections and biopsy ROIs. The sample-to-sample distribution variability was checked by exploring the low-dimensional multivariate representation of ROIs using principal component analysis (PCA). The inhouse code was implemented in Python using the scikit-learn library to explore the data distribution among all anatomical tissue regions, as well as the adenocarcinoma class solely.

### 2.5. Multivariate Modeling

For the overall class separation, PCA analysis with 1000 components was conducted followed by 3-dimensional LDA on all CRC cross section ROIs to explore the separability of spectral information among all 6 anatomical tissue regions. To explore the distribution shift in adenocarcinoma ROIs, an adversarial experiment was run to predict the patient from which the ROI data arose. PCA analysis with 1000 components followed by 1-dimensional LDA was used for this purpose. For recognition of the anatomical tissue region and to increase the generalizability of modeling, a leave-one-patient-out cross-validation experiment was implemented. In each step, the model was trained based on the ROIs of 9 patients, and then tested on the ROIs of the remaining patient as a prospective set. The overall prediction accuracy was calculated by accumulating the correct classification rate of test ROIs in the 10-fold scheme mentioned above. This experiment was conducted in 3 configurations: (1) a 6-class classification model using cross-section ROIs for both test and train, (2) a binary classification model for adenocarcinoma versus non-neoplastic ROIs from tissue cross sections for both test and train, and (3) a binary classification model for adenocarcinoma versus non-neoplastic ROIs from tissue cross sections as the training set, and corresponding biopsy ROIs for testing. When using the biopsy ROIs as a test set, ROIs from the corresponding cross section from the same patient were left out of the training set. For all the experiments, the models consisted of PCA with 1000 components, followed by LDA with dimensions equal to the number of classes minus one (Figure 1).

### 2.6. Univariate Modeling

To identify the prediction power (importance) of each individual m/z ratios via modeling, a LDA was conducted directly on each individual ion instead of multivariate PCA. A similar leave-one-patient-out cross-validation experiment was performed to increase the generalizability of the results. The m/z ratios were then sorted based on their prospective prediction power to identify the most significant ions. The experiments included (1) a binary classification model for LVI prediction, using cross-section ROIs for both test and train, (2) a binary classification model for sex, using cross-section ROIs for both test and train.

### 2.7. Statistical Analysis

A combination of univariate and multivariate statistical methods were used for data exploration and analysis. Using Metaboanalyst, volcano analyses were used to identify the m/z ratios that were the most differentiative between selected classes using the criteria of a fold change equal to or greater than 2 and a *p* < 0.001. For example, 2-class comparisons between pathological regions included adenocarcinoma versus all other non-neoplastic regions, and benign mucosa versus all other regions. In addition, the same analysis was used to compare patients across LVI status and biological sex. In the case of biological sex, in which the number of differences was significantly less; the criteria of a fold change greater than 1 and a *p* < 0.001 was used instead. The statistical analysis was compared to the prospective prediction power of the selected ions. As tandem MS-based characterization of ions was not performed in this study, the m/z identities of differentially abundant m/z ratios was based on a number of criteria; (1) By comparing m/z ratios to publicly available data bases such as the Human Metabolome Database (HMDB) and Metlin, (2) Comparison to m/z ratios specifically identified in other studies involving high-resolution mass spectrometry [19,20,21,22]. As m/z ratios can correspond to multiple species of complex lipids, m/z ratios were labelled on the basis of general lipid classes for the purposes of our feasibility study.

## 3. Results

### 3.1. Characterization of Small Molecule Profiles across Colorectal Tissue Subregions

**Figure 2:**
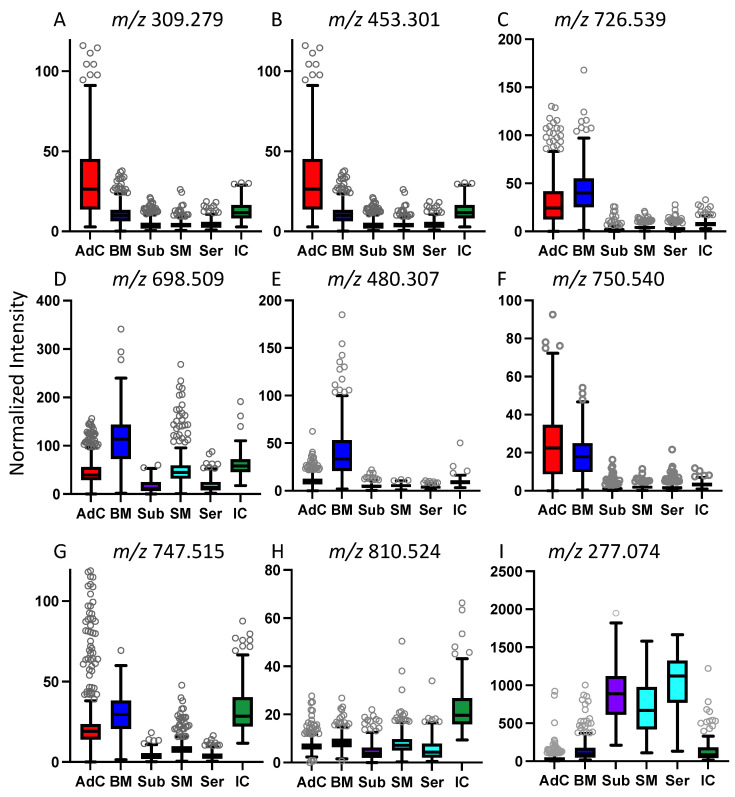
Differentially abundant ions. Boxplots present the differential abundance (TIC normalized intensity) of selected m/z (**A**–**I**) that are elevated in adenocarcinoma and other specific tissue regions. Characterisation of certain m/z ratios are presented in Table 2. Abbreviations: AdC—adenocarcinoma, BM—benign mucosa, Sub—submucosa, SM—smooth muscle, Ser—serosa, IC—inflammatory cells.

**Figure 3:**
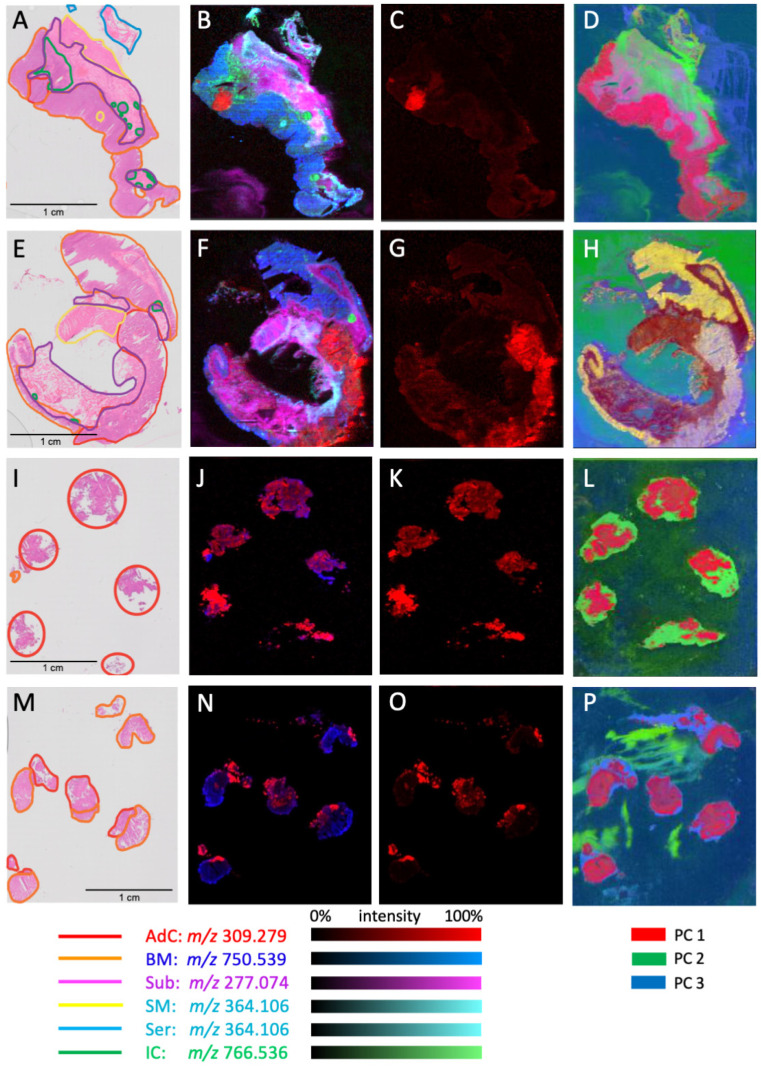
Histopathology annotation and corresponding mass spectrometry images of colorectal tissue. Hematoxylin and eosin (**E**,**H**) micrographs for representative colorectal cross sections (**A**,**E**) and biopsies (**I**,**M**), including annotation of adenocarcinoma (AdC) and other non-neoplastic tissue regions: BM—benign mucosa, Sub—submucosa, SM—smooth muscle, Ser: serosa, IC—inflammatory cells. Composite image of single-ion heatmaps featuring m/z ratios that are specifically abundant in the indicated region (**B**,**F**,**J**,**N**). Single-ion heatmap focusing on the relative abundance of m/z 309.279 which is spatially correlated with adenocarcinoma (**C**,**G**,**K**,**O**). Composite multivariate image based on the first three components of TIC-normalized pixels subjected to dimension reduction by PCA (**D**,**H**,**L**,**P**).

A total of 10 human CRC tissue specimens, randomly collected from patients undergoing surgical resection irrespective of tumour subtype, were used to generate paired fresh-frozen representative CRC cross sections, and biopsy samples including nontumour regions. A subset of the histopathologic features of these samples is summarized in Table 1. After DESI analysis, all fresh-frozen specimen sections were H and E stained and used to generate whole slide digital images, which were histologically annotated for tumour and nontumour regions, and compared with DESI small molecule profiles (Figure 1, Supplementary Figure S1). Using the histopathology annotations, regions of interest (ROI) associated with six histopathological regions were selected, including adenocarcinoma (AdC) cross section (*n* = 563)/biopsy (*n* = 551), benign mucosa (BM) cross section (*n* = 437)/biopsy (*n* = 309), submucosa (Sub) (*n* = 384), smooth muscle (SM) (*n* = 384), serosa (Ser) (*n* = 238) and inflammatory cells (IC) cross section (*n* = 141)/biopsy (*n* = 116) which served as the basis for univariate and multivariate analyses (Figure 1). Ions associated with the six anatomical regions were identified using a combination of the volcano and the supervised multivariate analyses. Figure 2 and Table 2 present the relative abundance of selected ions across histopathological regions, as well as possible identities. Prioritized ions were selected on the basis of a fold change >2 when compared to all other regions, and the *p*-values < 0.001. m/z 309.279 (gondoic acid) exhibited a 4.5-fold greater relative abundance in adenocarcinoma, as compared to all other anatomical regions. When using a leave-one-patient-out approach, concentrations of gondoic acid alone were able to correctly classify ROIs as either adenocarcinoma or other non-neoplastic tissue, 86% of the time. Accordingly, single ion heat map images of m/z 309.279 were spatially correlated with the histopathology-annotated adenocarcinoma regions in the colorectal tissue cross sections as well as the biopsies (Figure 3). Interestingly, seven other ions corresponding to long-chain (LCFA) and very-long-chain fatty acids (VLCFA) were found to be specifically localized to the adenocarcinoma regions, most of which exhibited fold-changes of 3–6, and achieved correct classification rates for identifying adenocarcinoma of up to 88% (Table 2). m/z 453.301 was also selectively abundant in adenocarcinoma and exhibited a high single-ion classification rate of 87% (Figure 2 and Figure S1).

**Figure 4:**
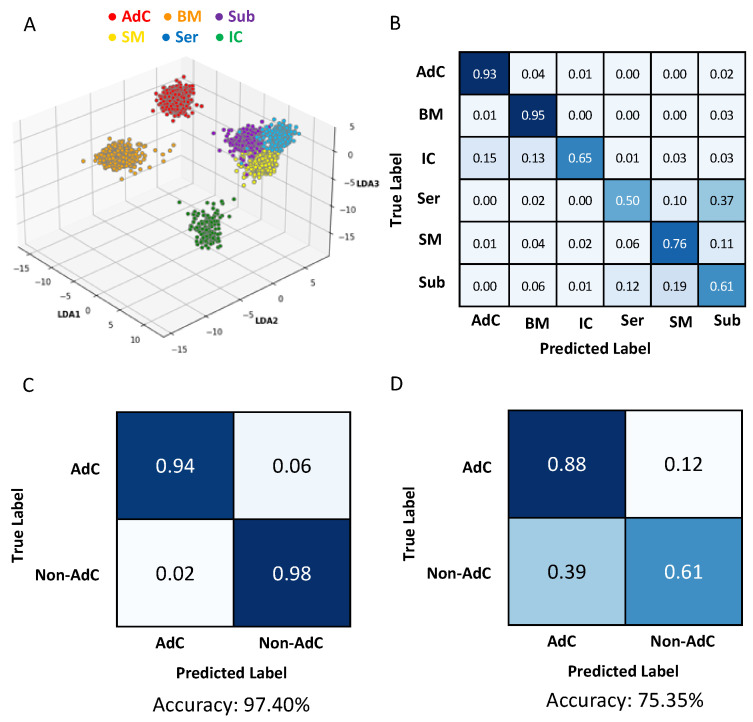
Multivariate analysis of CRC cross sections and biopsies. (**A**) A 3-dimensional plot of a 6-class PCA/LDA model using ROIs from tissue cross section. (**B**) Leave-one-patient-out cross validation of the 6 regions present in the tissue cross sections: adenocarcinoma (AdC), benign mucosa (BM), inflammatory cells (IC), serosa (Ser), smooth muscle (SM), and submucosa (Sub). (**C**,**D**) Confusion matrices for leave-one-patient-out cross validation using a binary PCA/LDA model constructed with the cross sections. The model was tested on ROIs from both the cross sections (**C**) as well as the biopsies (**D**).

While ions m/z 698.509 and m/z 726.539, corresponding to phosphatidylethanolamines (PE) were also highly abundant in adenocarcinoma, they were abundant in the benign mucosa as well. Other species that were more specific to benign mucosa included m/z 480.307 (Lyso PE) and m/z 750.540 (PEp), whose spatial distribution was in agreement with the histopathology annotation for benign mucosa, as shown in the two-dimensional heatmap in Figure 3. As colorectal cancer commonly develops in the epithelial cells of the mucosa, it was not surprising that certain ions were present in both of these regions. Interestingly, the histopathology analysis of one of our samples revealed two distinct tumour regions located either adjacent to the benign mucosa, or adjacent to smooth muscle/serosa (Figure S2). The impact of the juxtaposition of the two tumour regions on metabolite profiles was evident by unsupervised K-means clustering and PCA. The tumour ROIs formed two distinct clusters, one of which formed a single cluster together with the benign mucosal ROIs, while tumour ROIs adjacent to the muscle/serosa formed a separate cluster (Figure S2). Regions of inflammatory cells exhibited distinctly abundant ions corresponding to phosphatidylserines m/z 810.524 and m/z 812.535, as well as m/z 766.536, whereas considerable overlap was observed in concentrations of many ions abundant in serosa, smooth muscle and submucosa; including m/z 277.074, m/z 292.061, and m/z 364.106 which did not differ significantly across these three regions (Figure 4 and Table 2). This is consistent with the similar morphology associated with the cells in these tissue regions as both submucosa and serosa are mostly composed of loose connective tissue and have a similar histologic appearance. Whereas the muscularis is predominantly composed of smooth muscle cells and might be expected to have distinct mass spectral profiles, it is also a type of connective tissue that is embryologically derived from the mesoderm just like the serosa. When examining the ability of individual ions to predict all six tissue classes, ions such as m/z 750.540 and m/z 453.301, with notable abundance in adenocarcinoma and benign mucosa, achieved among the most favourable correct classification rates of 48 and 52% as they had distinct concentrations across the other tissue types as well (Table 2, Figure 2). Other ions with the highest correct classification rates across the six tissue types include m/z 752.548 and m/z 774.537 (PEp, 52%). Our observations were consistent with a PCA/LDA-based model, as adenocarcinoma, benign mucosa, and inflammatory cell ROIs exhibited distinct groupings, whereas serosa, smooth muscle, and submucosa displayed less variation between their mass-spectral profiles (Figure 4A). The model achieved 93% and 95% correct classification rates for identifying adenocarcinoma and benign mucosa, respectively (Figure 4B). Inflammatory cells only attained a 65% correct classification rate, in which misclassifications were due to the incorrect recognition of adenocarcinoma and benign mucosa, perhaps because colorectal cancer is an inflammation-related condition and inflammatory cells can be found both as normal components of benign colorectal tissue and in association with adenocarcinoma. Given the morphology-based resemblance between serosa, smooth muscle, and submucosal regions, the correct classifications for these pathological regions were expectedly reduced and most often misclassified within this triad. Since adenocarcinoma, benign mucosa, and inflammatory cells were only observed in our biopsy samples, it is anticipated that this result would not impact the clinical utility of our current methodology. A binary model containing adenocarcinoma vs. all non-tumour regions, achieved an accuracy of 97.40%, with a sensitivity and specificity of 94% and 98%, respectively (Figure 4C). When applying the binary model to the simulated biopsy samples, the overall accuracy of the model was 74.51%, owing to lower specificity (61%), while a sensitivity of 88% for adenocarcinoma was achieved (Figure 4D).

### 3.2. Patient-Based Stratification of Spectra from Regions of Colorectal Adenocarcinoma

Given that DESI is a nonquantitative technique, sample-to-sample variability was assessed using PCA across all of the colorectal tissue ROIs (*n* = 2152) (Figure S3A), as well as solely the adenocarcinoma ROIs (*n* = 563) (Figure S3B). Overall, the PCA plots exhibited a lack of separation among ROIs across all samples, suggesting that analytical variability achieved throughout the data acquisition was reasonable. A similar pattern was observed by plotting the adenocarcinoma ROIs independently of all other pathological regions (Figure S2B).

**Figure 5:**
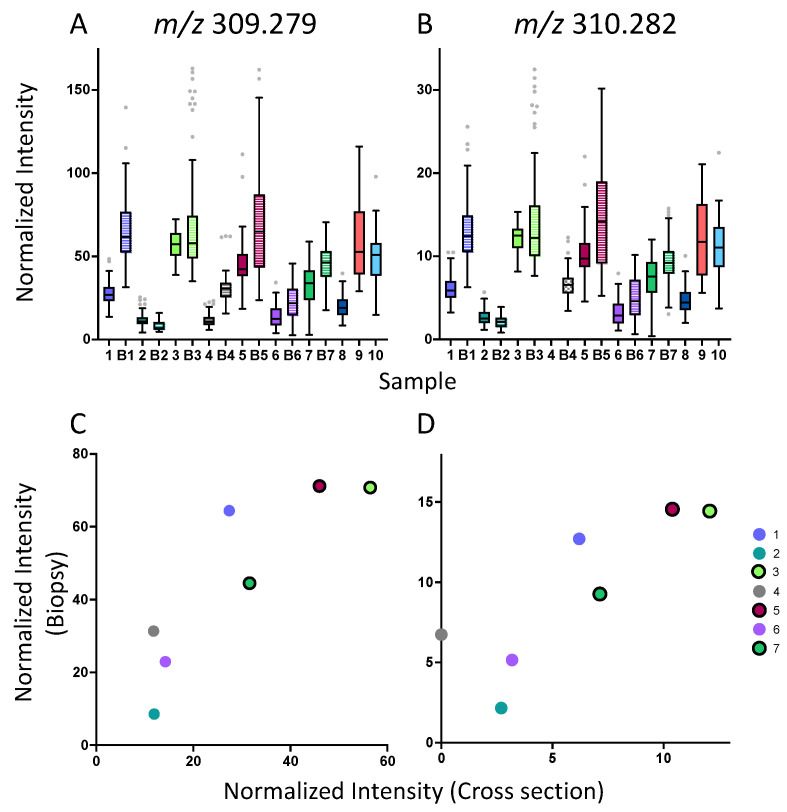
Comparison of gondoic acid levels in cross sections and biopsy specimens. (**A**,**B**) Boxplots depicting the TIC-normalized intensity of m/z 309.279 and 310.282 (Gondoic acid) in adenocarcinoma tissue regions across all colorectal tissue cross sections, in comparison with corresponding biopsies, where possible. m/z 310.282 is a ^13^C-containing isomer of m/z 309.279. (**C**,**D**) Scatter plots presenting the correlation of intensities of m/z 309.279 and 310.282 between cross sections and biopsies. Sample IDs are indicated by number, where biopsies corresponding to each cross section are denoted with ‘B’. Data points circled in black indicate LVI+ samples.

To test the biological variability of our samples, a 10-fold cross validation was performed by sample, in which three ions classified tumour ROIs by the patient with an average accuracy of ~40% including m/z 755.557, 726.539, and 310.282. While correct classification by patient is low, it does suggest that mass spectral profiles may be associated with common pathological characteristics among certain patients. There were four patients that experienced considerable overlap with each other. Patient two stood out as being the only subject with a mismatch repair (MMR) deficiency, whereas clinical profiles for the other patients (1,8,10) did not differ from other patients in the cohort; however, it should be noted that when all adenocarcinoma ROIs were plotted on the PCA, patients one and six exhibited slightly altered mass-spectral profiles. Interestingly, m/z 726.539 and 310.282 (or their related species) were initially found to differentiate adenocarcinoma from other pathological regions (Figure 2). m/z 309.279 was not selected as a significant feature for patient-based cross validation, however, its ^13^C isomer, m/z 310.282 was selected. When plotting the relative concentrations of m/z 309.279 and 310.282, a 5–7-fold difference among cross sections was found, with a similar pattern observed in the biopsies (Figure 5). When comparing m/z 309.279 and 310.282, similar relative intensities between these ions can be seen.

**Figure 6:**
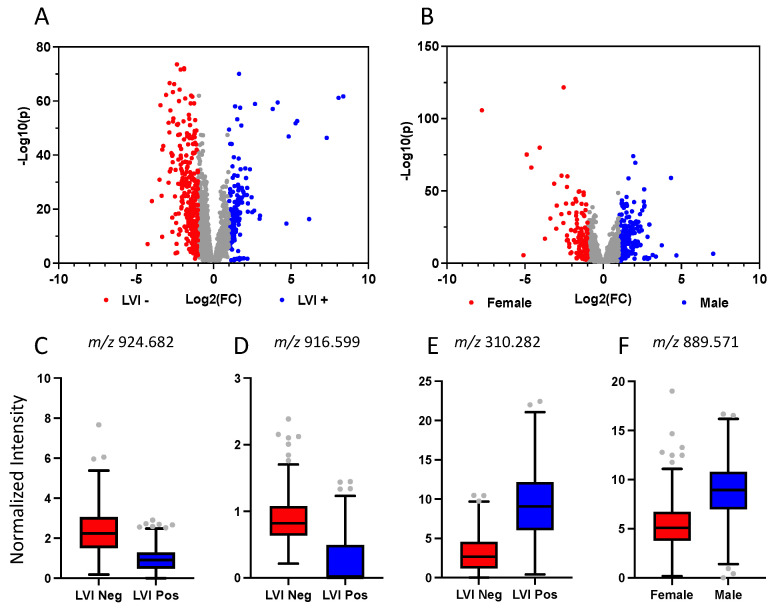
Stratification of DESI spectra by clinical features. Volcano plots revealing m/z ratios that differ significantly between LVI-negative and LVI-positive patients (**A**) and females and males (**B**) are shown. Relative abundance based on TIC-normalized intensities of specific ions that significantly differ by LVI status and biological sex are presented as boxplots (**C**–**F**). m/z 310.282 is the ^13^C isomer of gondoic acid.

Comparing lymphovascular invasion (LVI)-positive with LVI-negative samples, 5/6 LVI-positive patients were found to have elevated m/z 309.279 and 310.282 (Figure 6). Tumour ROIs were then labelled on the basis of LVI status to identify ions that may correlate with this prognostic feature. Firstly, a multivariate analysis of mass spectral profiles stratified by LVI status was attempted, however, this yielded poor accuracy (<60%). This was not surprising as only tumour ROIs were included in our LVI analysis, which were shown to be highly different from non-neoplastic regions (Figure 4). Use of individual ions was able to more accurately distinguish those with LVI compared to those without. Figure 6 and Table 3 present ions determined by volcano analysis that significantly differed with LVI status, including m/z 310.283 (^13^C gondoic acid), m/z 916.599 (oxidized PS), m/z 924.682 (oxidized PS/PC), and m/z 281.248 (oleic acid). In addition, m/z 909.627 (oxidized PG) and m/z 866.652 (PE) correctly identified LVI status using leave-one-patient-out cross validation, with 77.98% and 79.75%, respectively (Table 3).

Similarly, tumour ROIs were then stratified by biological sex, as it is another source of clinical variation and a risk factor for CRC. This is presented by the volcano analysis and boxplots of ions that significantly differentiated males and females (Figure 6 and Table 3). Ions such as m/z 426.364 (Carnitine) and m/z 799.685 (TG) were elevated in females, whereas, the relative intensity of ions such as m/z 889.571 were elevated in males.

## 4. Discussion

Using histopathology-validated spectra acquired by DESI, a greater than 95% classification accuracy was achieved for identifying colorectal adenocarcinoma, using a leave-one-patient out approach that also showed promise when applied to simulated colorectal biopsies, though with lower accuracies. Metabolic profiles could also be used to differentiate between certain anatomical regions of CRC tissue. The elevated abundance of ions corresponding to LCFAs and VLCFAs were among the most significantly increased species observed in CRC as compared to all other adjacent tissue types. When stratifying adenocarcinoma regions by LVI status, multiple oxidized phospholipids were found to be elevated in LVI-negative samples, revealing the potential diagnostic and prognostic applicability of DESI.

A number of studies employing DESI to profile colorectal cancer tissue have been conducted that show good discrimination between CRC and non-neoplastic tissue regions using multivariate models driven by the differential abundance of complex glycerophospholipids, which is altered in malignancy [23]. A reciprocal abundance of PEs m/z 726.54 and 750.54 in adenocarcinoma versus benign regions was shown in a pilot study involving one specimen [24]. Another study revealed a PEp (m/z 698.51) in combination with a series of PGs and PIs, such as m/z 747.52, were also characteristic of CRC tissue [22]. While all of these ions were detected in adenocarcinoma in our study, m/z 698.50 and m/z 726.53, were detected in benign mucosa as well, presumably since CRC arises in this anatomical region. Similarly, while m/z 747.51 was not determined to be specific to adenocarcinoma, this ion helped drive the PCA/LDA model that aimed to differentiate all anatomical regions explored [22]. This emphasizes conclusions from other CRC studies that individual biomarkers from tissue profiles may not accurately recognize tumor tissue, though relatively complex patterns of m/z ratios assessed by multivariate methods may be required.

In cells isolated from colon cancer tissue, Hofmanova et al., reported elevated lyso phospholipid-to-phospholipid ratios, specifically for LysoPEs and LysoPS species [23]. While Lyso PE (16:0) was not found to be significantly elevated, Lyso PE (18:0) was found to be significantly elevated. Counterintuitively, a Lyso PE with the same m/z (m/z 480.30) was observed to be elevated in benign mucosa. Our group previously observed these same two Lyso PEs as being abundant in benign prostate tissue, and concentrations were inversely proportional to the prostate cancer grade implying an increased utilization of lysophospholipid in hyperproliferative cell types [25]. Although various roles for Lyso PLs in tumorigenesis have been proposed, such as use of energy sources [26] and as signalling molecules via Lyso PL receptors [27], the specific mechanistic details on the role of lysophospholipids in colorectal cancer remain unclear; despite being identified as key discriminating features of colorectal cancer tissue in a number of studies.

Among our most striking observations was that at least eight LCFAs or VLCFAs featuring up to 26 carbons were specifically elevated in adenocarcinoma tissue. m/z ratios consistent with the four-step elongation of saturated stearic acid to cerotic acid, and the elongation of monounsaturated oleic acid to ximenic acid was found. De novo fatty acid synthesis occurs in tumour tissue, increasing the concentration of palmitic acid which can be further converted to a myriad of saturated and monounsaturated FAs via the action of fatty acid elongases and desaturases, which act as building blocks for complex glycerophospholipids used in the membranes of rapidly-proliferating cancer cells [28,29,30]. Enhanced expression of fatty acid synthase (FASN) has been observed in a number of cancer cell types and expression levels have been shown to be correlated to colon cancer progression and survival [31,32]. Furthermore, the delta-9-sterol CoA desaturase (SCD1), and the elongation of very long fatty acids protein one (ELOVL1) involved in the desaturation of stearic to oleic acid, and their respective elongation, have also been shown to be upregulated in colorectal cancer [30,33,34]. Using gas chromatography–mass spectrometry of extracted colorectal tissue, Mika et al., demonstrated increased concentrations of the same saturated and monounsaturated VLCFAs observed here, which rationalizes the increased expression of FA-metabolizing enzymes and the availability of substrate [33]. Another study also showed that these same VLCFAs were incorporated into Lyso PLs which were elevated in CRC tissue [23]. Conversely, the precursors stearic and oleic acids, were not significantly elevated in adenocarcinoma in this study, presumably since they were rapidly subjected to desaturation and elongation. Mika et al., also showed that cerotic acid could be detected in the circulation of CRC patients, though it remained undetectable in healthy controls, suggesting that tissue-based FA biomarkers of CRC could potentially serve as noninvasive blood tests to determine the presence of CRC, as well as monitor the response to treatment [33].

LVI is a well-established poor prognostic feature for CRC, indicative of increased metastatic risk, both to regional lymph nodes and more distant sites such as the liver. In addition to providing prognostic information, identification of LVI by the pathologist in a given tumour may influence adjuvant oncologic treatment decisions. As such, the metabolomic differences associated with LVI status was explored. Ions including m/z 916.59 (PS), 909.68 (PG), and 924.62 (PS/PC) were elevated in LVI-negative patients and represent oxidized phospholipids. Others have suggested that the oxidation of phospholipids stimulates its externalization to the outer cell membrane, leading to apoptosis [4,5]. This would indicate that patients experiencing LVI have a decrease in phospholipid oxidation compared to LVI-negative patients, therefore reducing active apoptotic mechanisms, which is a known hallmark of cancer [6,13,35]. As patients with LVI are typically given a worse prognosis, we propose that LVI may be associated with reduced, or lack of tumour cell death. While the observed increase of oleic acid (m/z 281.24) in LVI-positive patients was unexpected, levels of this FA may be confounded by increased de novo FA synthesis and downstream elongation. As the crosstalk between CRC tumour cells and adipocytes in the tumour microenvironment is a subject of ongoing investigation, it is reasonable to consider that a more advanced LVI-positive CRC may exhibit metabolic phenotypes favouring survival benefits from oleic acid consumption.

While there were limited differences in spectra associated with biological sex, our study implies that there may be subtle differences in CRC progression between biologic males and females. For example, females exhibited an increase in m/z 426.36 (carnitine) [36]. Carnitine is vital to cell metabolism as it shuttles acyl groups supplying energy through fatty acid oxidation [37]. Cai et al., observed elevated fatty acyl carnitine, such as palmitoylcarnitine and stearoylcarnitine in women with right-sided CRC, supporting increased fatty acid oxidation through carnitine shuttling to support tumour growth [38]. This work is consistent with this sex-specific difference in tumour energy production in CRC, however a larger cohort will be required to confirm these findings.

While our results are encouraging, a number of limitations should be acknowledged. An increased sample size with a broader profile of clinical and pathological parameters including tumour molecular subtypes and genomic status will enable the identification of additional differential metabolomic signatures. For example, given the known variation between colon and rectal cancers in terms of clinical parameters including treatment response, these cancers would be expected to have different metabolomic properties. Thus, future cohorts would ideally include a balanced number of colon and rectal samples to address this hypothesis. Additionally, in this small feasibility study, specific metabolite identities were not determined using first principles, as a number of mass spectrometry studies involving colorectal tissue with confirmed metabolite identities have already been completed [19,21,22]. However, metabolite identification, using tandem MS and/or ion mobility, is required to fully assess mechanistic insights in this study and help resolve complex lipids with identical masses. Our method of ROI selection precluded us from assessing field effects in the vicinity of the tumour, as nine-pixel ROI’s were used, that were uniformly positioned across anatomical regions rather than stratifying ROIs based on distance from the tumour. For example, the lack of differential arachidonic acid (AA) metabolites detected was somewhat surprising, given the inflammatory nature of CRC. One report revealed enhanced AA around the outer edges of CRC spheroids using MALDI-TOF [39]. Reproducing this observation statistically with our approach was not possible, though this effect was observed qualitatively. Furthermore, no morphological changes were noted by histopathology around the tumour perimeter, limiting our ability to validate these observations; pointing out that chemical changes do not always reflect morphology, which is the current gold standard. Given the potential clinical implications of this work, the speed of DESI data acquisition may be limiting. While related techniques such as I-Knife [21,40] and MassSpec Pen [41] are rapid, they sacrifice the spatial mapping of analytes, which is an advantage of DESI. One can envision complementary roles for these MS modalities in pathology and at the point of care.

Taken together, this study has demonstrated that DESI profiles align with histopathological and prognostic biomarkers in both CRC cross sections and biopsy samples. It provides biochemical insights into the molecular basis of CRC because of the spatially-correlated ions that can be rationalized in the context of biological pathways known to be altered in CRC. This gives confidence in profiling techniques such as DESI to put in practice the detection of biomarkers in more rapid, early screens, enhancing the information available to clinicians, and optimizing clinical decision making and outcomes for CRC patients.

## Figures and Tables

**Table 1:** Colorectal cancer patient cohort and clinical features.

Study ID	Sex	Age	Procedure	T. Site	T. Type	Grade	Size	pT ^1^	pN ^1^	pM ^1^	LVI ^2^	MMR ^3^
1	F	67	Right hemicolectomy	Cecum	Mucinous	G2	4.9	3	1a	N	N	Intact
2	F	73	Subtotal colectomy	Ascending	AdC ^4^	G2 (G3 20%)	9	3	0	N	N	Deficient
3	M	72	Rectosigmoid resection	Sigmoid	AdC	G2	6	3	0	N	Y	Intact
4	F	92	Right hemicolectomy	Cecum	AdC	G2	7	3	0	N	N	Intact
5	M	61	Right hemicolectomy	Hepatic flexure	AdC	G2	2.5	3	1a	N	Y	Intact
6	M	86	Right hemicolectomy	Ascending	Mucinous	G2	9	3	0	N	N	Intact
7	F	66	Low anterior resection	Rectum	AdC	G2	3.2	3	1a	N	Y	Intact
8	M	65	Right hemicolectomy	ICV ^5^	AdC	G2	7	3	0	N	Y	Intact
9	F	62	Right hemicolectomy	Ascending	AdC	G2	4	4a	2b	1c	Y	Intact
10	M	73	Sigmoid resection	Sigmoid	AdC	G2	6	3	0	N	Y	Intact

^1^ tumour staging post-resection; ^2^ lymphovascular invasion; ^3^ mismatch repair; ^4^ adenocarcinoma; ^5^ ileocecal valve.

**Table 2:** Differentially abundant ions in colorectal tissue.

m/z Bin	Measured m/z	Theoretical m/z	Delta (ppm)	Putative ID ^1^	Elevated in ^2^	Fold Change ^3^	Classification Rate (%)
309.279	309.2794	309.2799	1.6	FA(20:1) Gondoic	AdC	4.5	86 ^4^
337.331	337.3109	337.3112	0.9	FA(22:1) Euraic	AdC	5.4	86 ^4^
365.342	365.3419	365.3425	1.6	FA(24:1) Nervonic	AdC	6.6	<85 ^4^
393.373	393.3729	393.3736	2.3	FA(26:1) Ximenic	AdC	17.5	85 ^4^
311.295	311.2948	311.2956	2.6	FA(20:0) Arachidic	AdC	3.3	86 ^4^
339.326	339.3260	339.3269	2.7	FA(22:0) Behenic	AdC	2.6	<85 ^4^
367.357	367.3574	367.3582	2.2	FA(24:0) Lignoceric	AdC	3.9	87 ^4^
395.388	395.3884	395.3895	2.8	FA(26:0) Cerotic	AdC	4.3	88 ^4^
453.301	453.3014	n/a	n/a	Unknown	AdC	5.4	87 ^4^/53 ^5^
726.539	726.5402	726.5443	5.6	PE	AdC/BM	3.2	n/a
698.509	698.5094	698.5130	5.6	PEp	AdC/BM	3.7	n/a
480.307	480.3067	480.3096	6.0	Lyso PE	BM	5.5	n/a
750.540	750.5395	750.5443	6.4	PEp	BM	2.9	48 ^5^
752.548	752.5477	752.5600	16.3	PEp	n/a	n/a	52 ^5^
810.524	810.5245	810.5291	5.7	PS	IC	3.2	n/a
774.537	774.5371	774.5443	9.6	PEp	n/a	n/a	52 ^5^

^1^ FA—fatty acid, PEp—phosphatidylethanolamine plasmolagen, PS—phosphatidylserine; ^2^ AdC—adenocarcinoma, BM—benign mucosa, IC—inflammatory cells; ^3^ fold change in selected tissue region as compared with all other tissue regions, *p* < 0.001 for all comparisons; ^4^ correct classification rate based on the single ion indicated using binary classification (AdC versus all non-neoplastic tissue regions); ^5^ correct classification rate based on the single ion indicated, across six tissue type classes (AdC, BM, Sub, SM, Ser, and IC).

**Table 3:** Differentially abundant ions in samples stratified by lymphovascular invasion and biological sex.

m/z Bin	Measured m/z	Theoretical m/z	Delta (ppm)	Putative ID ^1^	Elevated in ^2^	Fold Change ^3^	Classification Rate (%) ^4^
916.599	916.5958	916.5921	8	Oxidized PS	LVI−	3.7	74
310.282	310.2825	310.2752	N/A	Gondoic acid ^5^	LVI+	3.1	75
924.682	924.6822	924.6699	13	Oxidized PC or PS	LVI−	2.6	76
909.627	909.6301	909.6226	5	Oxidized PG	LVI−	8.4	78
866.652	866.6491	866.6411	13	PE	LVI−	4.4	81
281.248	281.2483	281.2481	2	FA(18:1) oleic acid	LVI+	2	71
889.571	889.5688	889.5731	2	PA	M	1.6	71
799.658	799.6575	799.6588	1	TG	F	1.4	n/a
595.296	595.2935	595.2889	11	LysoPI	M	5.8	86
201.039	201.0392	201.0405	10	Succinylacetoacetate or Ethyl aconitate	F	4.6	n/a
426.364	426.3652	426.3589	13	Stearoylcarnitine or L-carnitine	F	4.9	66

^1^ FA—fatty acid, PS—phosphatidylserine, PC—phosphatidylcholine, PG—phosphatidylglycerol, PE—phosphatidylethanolamine, PA—Phosphatatic acid, TG—Triglyceride, PI—phosphatidylinositol; ^2^ LVI+/−—lymphovascular invasion positive or negative, M—male, F—female; ^3^ Fold change in selected tissue region as compared with all other tissue regions; ^4^ Correct classification rate based on the single ion indicated using binary classification for LVI status or biological sex; ^5 13^C-isomer of m/z 309.279 (gondoic acid).

## Data Availability

Data is contained within the article or supplementary material.

## Supplementary Materials

The following supporting information can be downloaded at: https://www.mdpi.com/article/10.3390/metabo13040508/s1, Figure S1: Histopathology annotation and mass spectrometry images of colorectal tissue; Figure S2: Altered mass spectral profiles between two adenocarcinoma regions in the same colorectal tissue cross section; Figure S3: Exploration of sample-to-sample variability of mass spectral profiles in colorectal tissue cross sections.
