# Supplementary material for: Metabolically Active Zones Involving Fatty Acid Elongation Delineated by DESI-MSI Correlate with Pathological and Prognostic Features of Colorectal Cancer
Source: Metabolites. 2023 Mar 31;13(4):508. doi: 10.3390/metabo13040508 (PMC10141897; doi:10.3390/metabo13040508)
Supplement: Supplementary file 1 [file metabolites-13-00508-s001.zip › metabolites-2274897-supplementary.pdf]

Metabolically active zones involving fatty acid elongation delineated by DESI-MSI correlate with pathological and prognostic features of colorectal cancer

CONTENTS

Figure S1. Histopathology annotation and mass spectrometry images of colorectal tissue.

Figure S2. Altered mass spectral profiles between two adenocarcinoma regions in the same colorectal tissue cross section.

Figure S3. Exploration of sample-to-sample variability of mass spectral profiles in colorectal tissue cross sections.

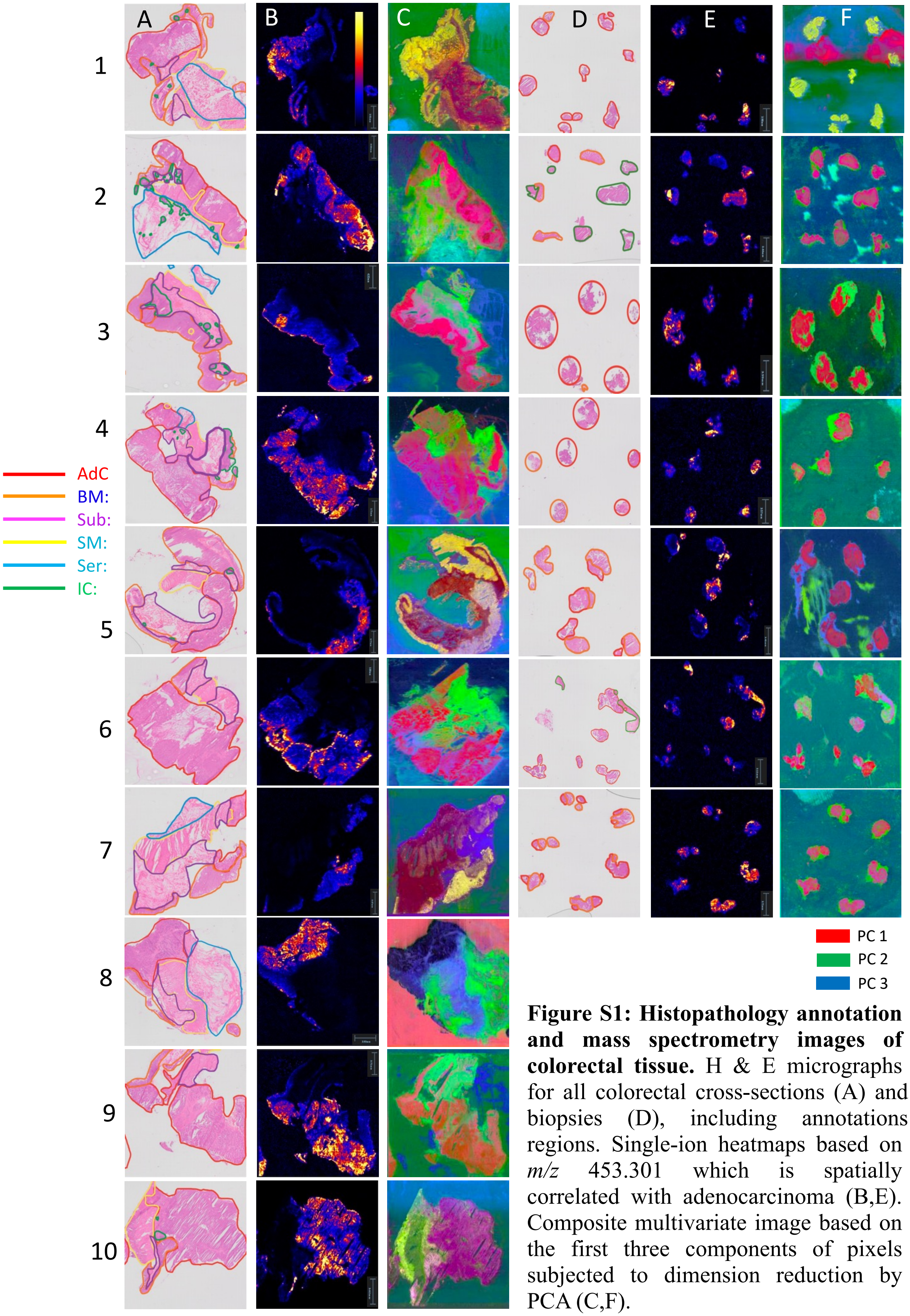

Figure S1: Histopathology annotation and mass spectrometry images of colorectal tissue. H & E micrographs for all colorectal cross-sections (A) and biopsies (D), including annotations regions. Single-ion heatmaps based on *m/z* 453.301 which is spatially correlated with adenocarcinoma (B,E). Composite multivariate image based on the first three components of pixels subjected to dimension reduction by PCA (C,F).

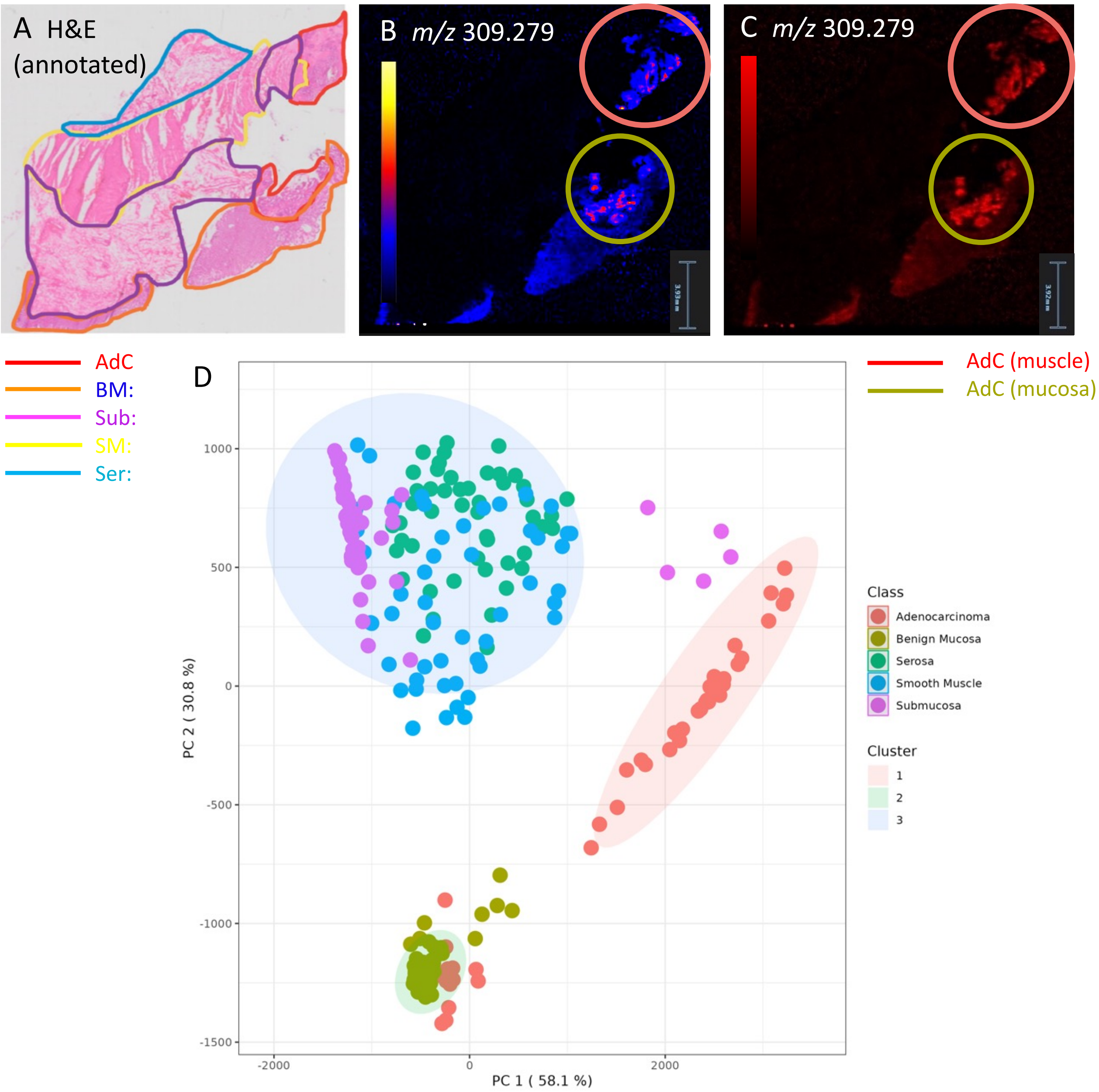

Figure S2: Altered mass spectral profiles between two adenocarcinoma regions in the same colorectal tissue cross section. One CRC tissue cross section had two regions of Adenocarcinoma (AdC), adjacent to mucosa, and smooth muscle (SM)/submucosa (Sub) (A). Both exhibited relatively high concentrations of *m/z* 309.279 (gondoic acid) (B,C). When All ROIs were analyzed by K-means clustering (Metaboanalyst) with n=3 clusters selected, the adenocarcinoma regions formed two distinct clusters consistent with their locations. The AdC region adjacent to mucosa, formed one cluster with benign mucosa, while the SM/Sub-associated AdC formed a second distinct cluster. All other tissue regions were grouped into a single cluster (D).

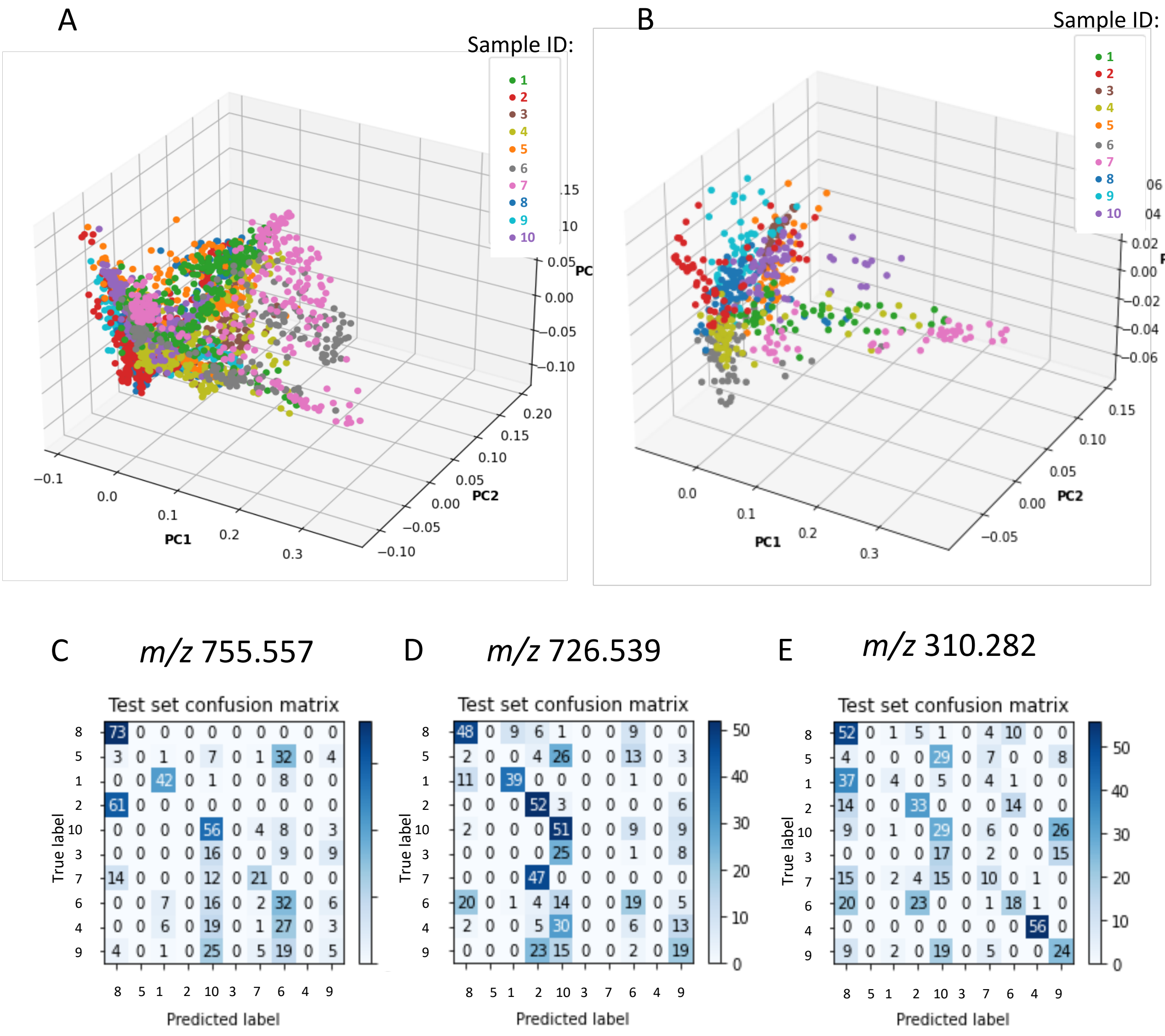

Figure S3: Exploration of sample-to-sample variability of mass spectral profiles in colorectal tissue cross sections. ROIs across all tissue regions in all cross sections are plotted in the PCA shown in (A) where ROIs are coloured by sample ID, presented in Table 1. The PCA in (B) shows the same analysis using the adenocarcinoma ROIs only. Panels C,D and E present confusion matrices for the top three ions that most accurately (40%) identified the correct sample.
